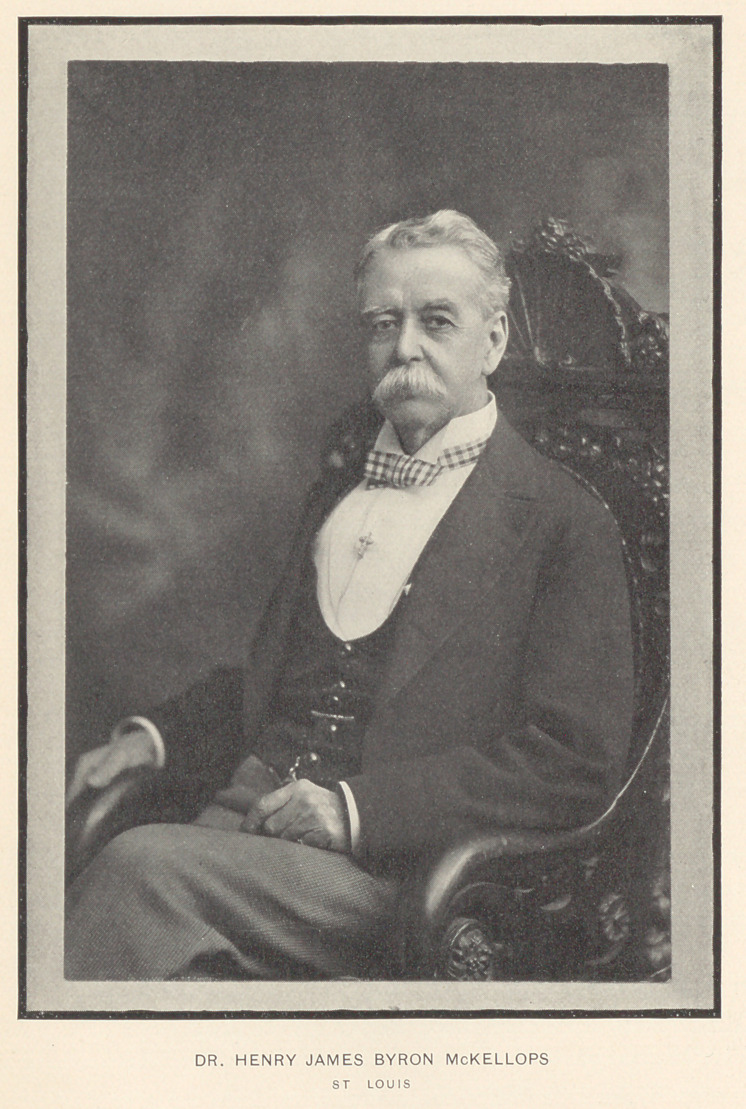# Henry J. McKellops, D.D.S.

**Published:** 1901-06

**Authors:** 


### Obituary.

### HENRY J. McKELLOPS, D.D.S.

Dr. H. J. McKellops died at his residence, St. Louis, Mo., April 23, 1901. He was born in the State of New York, at Saline, near Syracuse, on the 31st day of August, 1823. His father, James McKellops, died when the son was yet in his tender years.

As early as the year 1840 young McKellops went west with his mother and sister to the city of St. Louis. His education began in the city schools. Being a very active and intelligent lad, he had no difficulty in obtaining a position as messenger boy in the Missouri Legislature. In this employment Dr. McKellops made his first money, and proceeded at once to invest this in tuition at the Missouri State University, at Columbia, to which place his mother moved and remained for two years, while her son applied himself to his studies. The family returned to St. Louis in the year 1844, when young McKellops took a course of book-keeping in the Mercantile College. On the completion of this course, through kindly assistance, the young student secured a place in the city register's office.

About this time Dr. McKellops was attracted by the study of medicine and dentistry. He took a course of medicine, in the years 1846 and 1847, at the old St. Louis Medical College on Washington Avenue; but, through the persuasion of his brother-in-law, who was a dentist, he was drawn to the profession and practice of dentistry, and in a very short time, owing to his natural ingenuity and love of mechanical arts, became an expert operator. He opened his first office in Captain Elihu H. Shepard's building on Fourth Street, and at once stepped to the front rank of his profession.

In 1855 the Ohio Dental College conferred the degree of D.D.S. on him, and in 1864 he introduced the use of the mallet in London and Paris. It is hardly necessary to follow Dr. McKellops through his long and successful career in the city of St. Louis. His skill as an operator was early recognized, and secured him the patronage of the first families of the city. His fame extended outside of the city, through the State, and eventually through all the States of the Union. He was studious in his profession, inventive in his practice, always keeping to the front in whatever step of progress was made in dental surgery. He gave much of his time to the organization of Dental Societies and Associations, in the proceedings of which he always took a leading part. He was president of the Western Dental Association, first president of the Missouri State Dental Association, afterwards in turn becoming president of the American Dental Association and the Southern Dental Association. He was president of the St. Louis Dental Society. The selection of Dr. McKellops for all these important positions gives the best indication of his standing in his profession at home and abroad.

Dr. McKellops also has a military side to his career. He was elected captain of the St. Louis Cadets, an organization of young men commissioned by Governor Edwards in the year 1843. In 1845 he was made captain in the St. Louis Legion, and in 1846 commanded the Morgan Riflemen in the six months' service of the Legion in the Mexican War, making the noted six-months' expedition under Colonel Alton R. Easton, going south on the steamer "Conway" to New Orleans, thence to Brazos, Santiago, and up the Rio Grande to Matamoras. At the organization of the famous St. Louis National Guards in the fifties he enrolled himself as a member, and saw his first service in the riots of those days. Afterwards, in 1858, as assistant adjutant-general of the First Brigade, First Division, of Missouri Volunteers, he marched across the State with the expedition under General D. M. Frost, to put down the invasion of "Bushwackers" and "Jayhawkers" who were ravaging the western counties of Missouri.

Dr. McKellops was married on the 1st day of April, 1849, to Miss Annie Gower, of Tennessee, to whom eight children, five sons and three daughters, were born. Five of these survive, four sons and one daughter.

Dr. McKellops's labor in dentistry consisted principally in associative effort. In this, however, he did a great work. No man, among its many members, was better known or was more energetic in furthering the interests of the American Dental Association. No matter how far the meeting was held from his home, Dr. McKellops was always present; and when the National Dental Association was formed upon the destruction of the American, he transferred his interest to that body. He attended the meeting of that organization at Old Point Comfort in 1900, but it was evident, to the sorrow of his friends, that the energy that had helped to make these annual gatherings scientifically and socially delightful was no longer there, and that soon we would be forced to write, It is finished.

His aim was always for the highest in practice, and it is believed that he had, probably, few, if any, superiors as an operator. His poor health forced him out of practice some six months before death came to his relief. He was fully conscious that the end was near, and so stated to a friend who called on him two days before his final departure.

His funeral took place under the care of the St. Louis Lodge of Elks, the honorary pall-bearers being Drs. G. A. Bowman, E. H. Angle, Emma E. Chase, and A. H. Fuller.

## Figures and Tables

**Figure f1:**